# Cocreating the Visualization of Digital Mobility Outcomes: Delphi-Type Process With Patients

**DOI:** 10.2196/68782

**Published:** 2025-05-09

**Authors:** Jack Lumsdon, Cameron Wilson, Lisa Alcock, Clemens Becker, Francesco Benvenuti, Tecla Bonci, Koen van den Brande, Gavin Brittain, Philip Brown, Ellen Buckley, Marco Caruso, Brian Caulfield, Andrea Cereatti, Laura Delgado-Ortiz, Silvia Del Din, Jordi Evers, Judith Garcia-Aymerich, Heiko Gaßner, Tova Gur Arieh, Clint Hansen, Jeffrey M Hausdorff, Hugo Hiden, Emily Hume, Cameron Kirk, Walter Maetzler, Dimitrios Megaritis, Lynn Rochester, Kirsty Scott, Basil Sharrack, Norman Sutton, Beatrix Vereijken, Ioannis Vogiatzis, Alison Yarnall, Alison Keogh, Alma Cantu

**Affiliations:** 1 Population Health Sciences Institute Faculty of Medical Sciences Newcastle University Newcastle Upon Tyne United Kingdom; 2 School of Clinical Medicine Department of Public Health and Primary Care University of Cambridge Cambridge United Kingdom; 3 Translational and Clinical Research Institute Faculty of Medical Sciences Newcastle University Newcastle Upon Tyne United Kingdom; 4 NIHR Newcastle Biomedical Research Centre Newcastle University and The Newcastle Upon Tyne Hospitals NHS Foundation Trust Newcastle upon Tyne United Kingdom; 5 Robert Bosch Society for Medical Research Stuttgart Germany; 6 Digital Geriatrics Unit Medical Centre University of Heidelberg Heidelberg Germany; 7 Mobilise-D Patient and Public Advisory Group Newcastle Upon Tyne United Kingdom; 8 School of Mechanical, Aerospace and Civil Engineering University of Sheffield Sheffield United Kingdom; 9 Insigneo Institute for Silico Medicine University of Sheffield Sheffield United Kingdom; 10 Department of Neuroscience and Sheffield NIHR Translational Neuroscience BRC Sheffield Teaching Hospitals NHS Foundation Trust Sheffield United Kingdom; 11 Sheffield Institute for Translational Neuroscience University of Sheffield Sheffield United Kingdom; 12 The Newcastle Upon Tyne Hospitals NHS Foundation Trust Newcastle Upon Tyne United Kingdom; 13 Division of Clinical Medicine University of Sheffield Sheffield United Kingdom; 14 Department of Electronics and Telecommunications Politecnico di Torino Turin Italy; 15 Insights Centre Data Analytics University College Dublin Dublin Ireland; 16 School of Public Health Physiotherapy and Sports Science University of College Dublin Dublin Ireland; 17 Barcelona Institute for Global Health Barcelona Spain; 18 Department of Medicine and Life Sciences Pompeu Fabra University Barcelona Spain; 19 Network in Epidemiology and Public Health Center for Biomedical Research Madrid Spain; 20 McRoberts BV The Hague The Netherlands; 21 Department of Molecular Neurology University Hospital Erlangen Erlangen Germany; 22 Fraunhofer Institute for Integrated Circuits IIS Erlangen Germany; 23 Department of Neurology University Medical Center Schleswig-Holstein Campus Kiel Germany; 24 Sagol School of Neuroscience and Department of Physical Therapy Faculty of Medical and Health Sciences Tel Aviv University Tel Aviv Israel; 25 Rush Alzheimer’s Disease Center and Department of Orthopaedic Surgery Rush University Medical Center Chicago, IL United States; 26 Center for the Study of Movement, Cognition, and Mobility Neurological Institute Tel Aviv Sourasky Medical Center Tel Aviv Israel; 27 School of Computer Science Newcastle University Newcastle Upon Tyne United Kingdom; 28 Department of Sport, Exercise and Rehabilitation Northumbria University Newcastle Upon Tyne United Kingdom; 29 Department of Neuromedicine and Movement Science Norwegian University of Science and Technology Trondheim Norway; 30 School of Medicine Trinity College Dublin Dublin Ireland

**Keywords:** mobility, data visualization, wearable devices, digital mobility outcomes, cocreation

## Abstract

**Background:**

Recent technological advances in wearable devices offer new potential for measuring mobility in real-world contexts. Mobilise-D has validated digital mobility outcomes to provide novel outcomes and end points in clinical research of 4 different long-term health conditions (Parkinson disease, multiple sclerosis, chronic obstructive pulmonary disease, and proximal femoral fracture). These outcomes also provide unique information that is important to patients; however, there is limited literature that explores the optimal methods to achieve this, such as the best way to visualize patients’ data.

**Objective:**

This study aimed to identify meaningful outcomes for each condition and how to best visualize them from the perspective of end users.

**Methods:**

Using a Delphi-type protocol with patients as subject matter experts, we gathered iterative feedback on the cocreation of visualizations through 3 rounds of questionnaires. An open-ended questionnaire was used in round 1 to understand what aspects of mobility were most influenced by their health condition. These responses were mapped onto relevant digital mobility outcomes and walking experiences and then prioritized for visualization. Using patient responses, we worked alongside researchers, clinicians, and a patient advisory group to develop visualizations that depicted a week of mobility data. During rounds 2 and 3, participants rated usefulness and ease of understanding on a 5-point Likert scale and provided unstructured feedback in comment boxes for each visualization. Visualizations were refined using the feedback from round 2 before receiving further feedback in round 3.

**Results:**

Participation varied across rounds 1 to 3 (n=48, n=79, and n=78, respectively). Round 1 identified important outcomes and contexts for each health condition, such as walking speed and stride length for people with Parkinson disease or multiple sclerosis and number of steps for people with chronic obstructive pulmonary disease or proximal femoral fracture. The consensus was not reached for any visualization reviewed in round 2 or 3. Feedback was generally positive, and some participants reported that they were able to understand the visualization and interpret what the visualization represented.

**Conclusions:**

Through the feedback provided and existing data visualization principles, we developed recommendations for future visualizations of mobility- and health-related data. Visualizations should be readable by ensuring that large and clear fonts are used and should be friendly for people with vision impairments, such as color blindness. Patients have a strong understanding of their own condition and its variability; hence, adding additional factors into visualizations is recommended to better reflect the nuances of a condition. Ensuring that outcomes and visualizations are meaningful requires close collaboration with patients throughout the development process.

## Introduction

### Background

Mobility refers to the ability to move freely and easily to carry out activities of daily living and refers to movement in all forms, from moving out of a chair to walking [1]. It is necessary for daily tasks, participating in social activities, and maintaining independence [2]. Mobility is a meaningful aspect of health for individuals across multiple health conditions and plays a large role in their physical, social, and psychological experience [3]. In conditions where motor symptoms cause functional impairment, understanding an individual’s walking, posture, and how they move is important for measuring and monitoring condition severity [4]. Specifically, walking is an important clinical measure whereby factors such as walking speed and stride length can predict functional decline and increase risk of frailty and mortality [5,6]. Hence, it is essential to be able to measure walking accurately and meaningfully convey these data to patients to provide insights into progress and changes in their condition over time.

Recent technological advances in wearable devices (such as body-worn sensors) offer new potential for measuring mobility continuously in real-world contexts (collectively termed digital mobility outcomes [DMOs]). This in turn presents the opportunity for novel insights into aspects of health that are important to patients, such as their real-world walking speed and daily step count [7]. Crucially, providing individuals with access to, and insights from, their own data from such devices is linked to patient autonomy, enhanced health professional-patient communication, and improved compliance with physician recommendations through the enhanced trust between patients and those involved in their care [8-12]. However, the effectiveness of this feedback hinges upon the meaningfulness of the outcome to patients, particularly whether their symptoms and symptom burden can be reflected by the outcome and be interpreted by patients [13-15].

Integration of the wearable devices into health care systems presents significant challenges in the visualization of the complex data and the insights these generate [16]. Mobility can be affected by multiple factors, such as changes in symptoms, environmental factors, and medication, which creates challenges and added complexity when communicating insights from the data [17-19]. Hence, it is essential to ensure that data visualizations are both accessible and understandable to both patients and clinicians and reflect the nuances of patients’ mobility while also representing meaningful aspects of their health [14,20,21]. Creating visualizations that enable an individual to come to a full understanding of a person’s overall health condition can be challenging; hence, several approaches have been attempted without one prevailing approach [19]. In previous visualizations from wearable devices, a range of traditional visualization approaches, such as radar plots, bar charts, line plots, and box plots, have been used, as well as more novel approaches, such as a calendar depicting 6 months of data in 1 visual [21,22]. Consequently, exploring data visualization preferences is important yet nuanced, and the creation of the visualizations requires an understanding of the patient experience.

### Objectives

This work builds upon the efforts of the Mobilise-D consortium to validate DMOs and provide novel insights into several long-term health conditions, including Parkinson disease (PD), multiple sclerosis (MS), chronic obstructive pulmonary disease (COPD), and proximal femoral fracture (PFF) [17,23-25]. Research has been carried out to establish the technical, clinical validity, and acceptability of wearable devices and the analytic pipelines applied to quantify DMOs [17,23,26]. An additional part of ensuring implementation of the methods and outcomes is to determine how best to visualize DMO data for patients. Consequently, this study aimed to explore patient visualization preferences by (1) determining which DMOs are meaningful to individuals living with diverse long-term health conditions and (2) identifying how to best visualize them from the perspective of end users [12].

Previous research on wearable device data visualization has highlighted the lack of consistent preferences regarding how best to visualize the data. Several approaches have explored the use of varying levels of granularity, with some studies attempting to visualize a day and others 6 months [21,22]. While this was highly variable depending upon the outcome, for mobility it is common to use a week of data [18,19,21]. A major point of interest in the literature was embedding contextual data within visualizations, particularly in relation to clinical use to enhance the inferences made [18,19]. Condition-related information, such as symptoms, emotions, and medications, can influence the lived experience, and the data from wearable devices should be able to reflect this, as well as the time and location of activities [27,28]. While we know specific design approaches and elements are key to the creation of comprehensive visualizations, it is also important to consider the individualized nature of preferences when developing meaningful visualizations [12]. Hence, this study aimed to map DMOs into the lived experiences of mobility and symptom burden and explore the visualization preferences of patients using a week of DMO data.

## Methods

### Study Design

This study design was based on a Delphi methodology in which patients acted as subject matter experts to iteratively provide feedback on the cocreation of data visualizations through 3 rounds of questionnaires. It was designed in collaboration with academic researchers of Mobilise-D and the members of the Mobilise-D Public and Patient Advisory Group (PPAG) [29].

**Figure 1:**
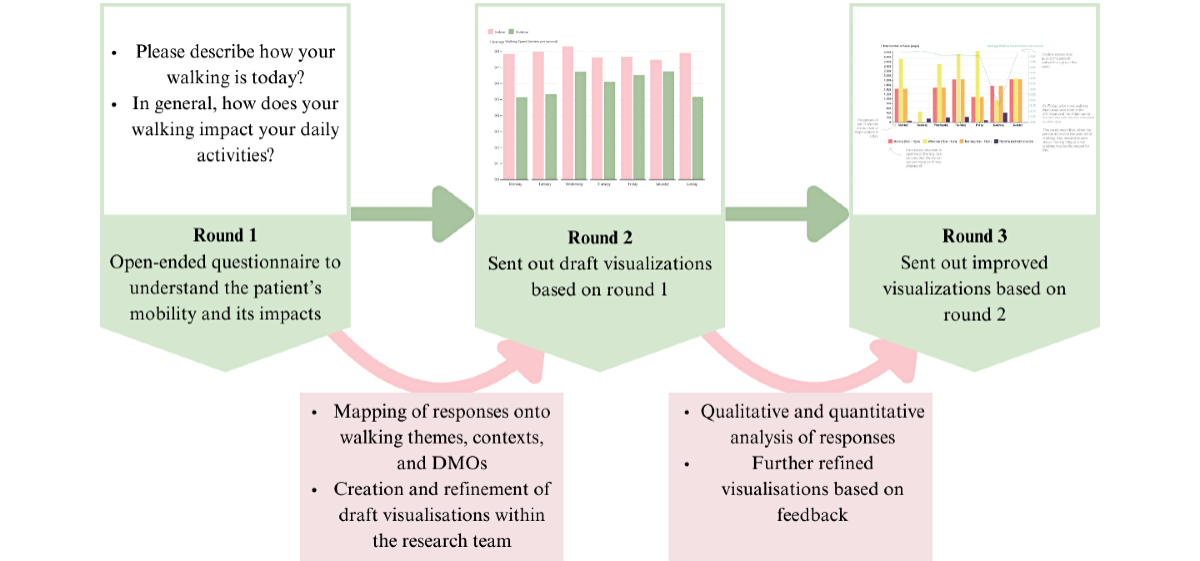
Outline of the study design, showing how the results from each round built upon each other. DMO: digital mobility outcome.

In total, 3 rounds were developed to explore the impact of mobility and symptoms and how DMOs can be visualized. A Delphi methodology was decided upon as it allows us to reach a consensus of patient preferences with an iterative, anonymous, multistage approach with controlled feedback of comments and scores on a 5-point Likert scale [30,31]. The Delphi methodology recommends 3 rounds of feedback; the first round is to generate qualitative data on a topic and the remaining rounds are to gain a consensus through Likert scales [31]. We followed the recommended 3 round; however, the methods used here will be described as a “Delphi-type process” due to deviations from Delphi-specific design elements. A key aspect of a Delphi methodology is ensuring participants completed previous rounds; however, due to ethical requests, this was not possible within this study [31,32]. This process involved a collaboration between patient feedback and researcher refinement, as described in Figure 1.

### Visualization Data

The data visualized in this study were collected as part of the Mobilise-D technical validation study (TVS) [23]. Participants with long-term health conditions wore a lower back wearable device (either a McRoberts MoveMonitor+ or the Axivity AX6) to provide real-world monitoring data for 7 consecutive days. Accelerometry data were collected and processed using the algorithms validated by Mobilise-D to produce DMOs [33]. In addition, GPS data were collected for participants, which allowed the data to be categorized into indoor versus outdoor. No activity or medication diaries were collected, which limited the contextual information available when visualizing the data.

### Public and Patient Involvement

This research was designed with members of the Mobilise-D PPAG [29]. In total, 4 international representatives (Belgium, Israel, Italy, and the United Kingdom) from the PPAG (2 with PD, 1 with a previous hip fracture, and 1 with MS) worked with academic researchers to develop each round of questions and visualizations to ensure they were appropriately worded and constructed and that the conclusions drawn from the data in each round were meaningful. Due to the international and remote work involved in the PPAG, perspectives of the digitally excluded unfortunately could not be included with the team.

### Recruitment

People with PD, MS, COPD, or a previous hip fracture primarily from Europe (British, Irish, Norwegian, and German) were invited to participate in this web-based survey Delphi-type study. Patients were purposively recruited from newsletters sent out to various patient charities and organizations (including Voice, MS Society, and Parkinson’s UK), social media, and through existing Mobilise-D clinical sites. A sample size of between 10 and 15 people per health condition was proposed in line with guidance for Delphi studies [31].

There were 239 responses to this initial form, which was then used as the distribution list for all rounds of the study. However, because of this method of recruitment, participants were unable to be individually tracked or compared per round, and this meant the participation of individuals in previous rounds could not be confirmed, which is not typical for a Delphi methodology [32]. Data were collected between June 2023 and June 2024. As this study aimed to iteratively build upon previous findings, the lack of participation in previous rounds may have impacted the participant’s understanding of the context surrounding each round. However, each round started with a summary of the project and the findings from previous rounds to mitigate some of this confusion.

### Data Collection and Analysis

#### Round 1

An open-ended questionnaire was used in round 1 to understand what aspects of mobility were most influenced by their long-term health condition (Multimedia Appendix 1). Questions were based on existing literature relating to how people with long-term health conditions experience mobility, specifically how it impacts them physically, socially, and psychologically [3]. This questionnaire was piloted with the PPAG before distribution, and the feedback led to questions being accompanied by a sample text written by the PPAG, exemplifying their mobility experiences and prompting participants (Multimedia Appendix 1).

The results of round 1 were aggregated and then analyzed using a deductive content analysis by 6 researchers with experience in digital mobility and qualitative research (AK, LD-O, AC, HG, CW, and KS). Deductive analysis was conducted by mapping responses onto walking themes, symptoms, and the most relevant DMOs (Table 1 and Multimedia Appendix 2). Responses from the questionnaire were discussed within the team regarding how elements of walking may be impacted by symptoms and how this may manifest within a DMO. This mapping involved the research team (including members of the PPAG and clinical experts—LR, GB, TGA, KvdB, and NS) discussing how elements of walking may be impacted by symptoms and how this might manifest within a DMO.

**Table 1:** Digital mobility outcomes used to map the patient experience during round 1, adapted from Mazzà et al [23] and Kluge et al [34].

Digital mobility outcomes	Description
Step count	Number of steps taken per day.
Number of WBs^a^	Number of WBs taken per day. A WB is a walking sequence containing at least 2 consecutive strides of both feet. The start and end of a WB are determined by a resting period or any other activity (nonwalking period).
WB duration	Duration between the start and the end of a WB. Defined as the daily average measured across each WB.
Stride length	The interval between 2 successive initial contacts of the same foot. Thereby, every stride contains 2 steps. Defined as the daily average measured across each WB.
Walking speed	Distance covered by the whole body within a certain time interval per unit time of walking. It is measured in meters per second and is the magnitude of the velocity vector. Defined as the daily average measured across each WB.
Step duration	Duration between an initial contact and the next initial contact of the opposite foot. Defined as the daily average measured across each WB.
Cadence	The number of steps taken per minute. Defined as the daily average measured across each WB.

^a^WB: walking bout.

In total, 3 rounds of cross-checking were conducted to clarify differences and determine specific coding rules before finalizing an agreed set of walking experiences deemed most important and how they map onto specific DMOs. Specific elements of walking most frequently mentioned were subsequently identified for each health condition to prioritize the aspects that were most important to patients. Following this, DMOs were identified that required the development of visualizations that addressed the most frequent walking experiences (Multimedia Appendix 2).

#### Round 2

The results of round 1 were used to develop draft visualizations that depicted a week of mobility data. Using the data from the Mobilise-D TVS, visualizations were drafted (Multimedia Appendix 3) [23]. Specifically, visualizations were prioritized based on the commonly listed experiences of mobility for each long-term health condition from round 1. The order of visualizations that were presented to participants was randomized to prevent bias.

On the basis of a list of relevant options provided by a visualization expert (AC), the research team and PPAG (AK, HG, CK, LD-O, JL, AC, NS, and TGA) discussed and voted for visualizations and contexts that they thought were most appropriate and useful for each element of walking. These decisions were reviewed, and the final visualizations for each element of walking were selected. The draft visualizations were then matched to a sample of text to explain why they were chosen and how the researchers felt that they related to people’s experiences (Multimedia Appendix 1).

Following the guidance from Trevelyan and Robinson [31], patients were asked to rate the usefulness and ease of understanding of each individual visualization using a 5-point Likert scale (0=not useful at all or very difficult and 5=very useful or very easy), as well as to suggest potential changes to the draft visualization. We considered consensus was reached when at least 75% of participants rated a visualization as ≥4 on the Likert scale [30]. In addition, qualitative responses were reviewed for relevant feedback, and a content analysis of responses was conducted by 2 researchers (JL and AK) to suggest changes for the visualizations.

#### Round 3

Using the results of round 2, the draft visualizations were revised with the aim of improving them in line with patient feedback. Revised visualizations were discussed between the academic research team and PPAG (CK, HG, LD-O, AK, JL, AC, NS, and TGA) and were then presented to patients alongside the justification for their creation (Multimedia Appendix 1). The order of visualizations that were presented to participants was randomized to prevent bias. Participants rated the revised visualizations on a 5-point Likert scale and provided comments, which were analyzed using a content analysis.

### Ethical Considerations

Ethics approval was granted by Newcastle University (2514/30280). To ensure anonymity in line with ethical requests, participants were asked to complete an anonymous form whereby their email address was collected and stored securely separate from demographic and response data from the study. These email addresses were stored securely on a password-protected computer and only accessed by researchers involved in questionnaire distribution. This was then used to send participants a link to take part in the study anonymously. Participation was voluntary, and responses were anonymous, with no personally identifiable data collected. All study participants received an information sheet about the study and were provided with the researcher’s emails in case of any questions. Participants were informed that the study should not take any longer than 10 minutes and were assured that they could withdraw without consequence at any time during the study. All participants gave informed consent. There was no compensation for participation in this study.

## Results

### Participants

Participation was dependent upon a round being fully completed; the demographics of participants across the 3 rounds are presented in Table 2. Participation varied across rounds 1 to 3 (n=48, n=79, and n=78, respectively). Across all rounds and participants, 63.4% (n=130) were female participants, 29.8% (n=71) of participants were below degree level or preferred not to say, 94.1% (n=193) were White and 37.6% (n=77) wore a wearable device daily. Considering that the sample size for COPD and PFF was lower than expected, their qualitative responses were included in the analysis but were not considered to have reached consensus [31].

**Table 2:** Participant demographics across all 3 rounds separated by each condition.

Demographic details			Round 1 (n=48), n (%)									Round 2 (n=79), n (%)									Round 3 (n=78), n (%)								
			PD^a^ (n=16)		MS^b^ (n=25)		COPD^c^ (n=1)		PFF^d^ (n=6)		PD (n=52)			MS (n=24)		COPD (n=1)		PFF (n=2)		PD (n=54)			MS (n=21)			COPD (n=2)		PFF (n=1)	
**Sex**																													
	Male	7 (44)		20 (80)		2 (100)		3 (50)		25 (48)			3 (13)		0 (0)		2 (100)		25 (46)			2 (10)		0 (0)			1 (100)		
	Female	9 (56)		5 (20)		0 (0)		3 (50)		27 (52)			20 (83)		1 (100)		0 (0)		29 (54)			19 (91)		1 (50)			0 (0)		
	Prefer not to say	0 (0)		0 (0)		0 (0)		0 (0)		0 (0)			1 (4)		0 (0)		0 (0)		0 (0)			0 (0)		0 (0)			0 (0)		
**Age (y)**																													
	18-30	0 (0)		1 (4)		0 (0)		0 (0)		1 (2)			2 (8)		0 (0)		0 (0)		0 (0)			1 (5)		0 (0)			0 (0)		
	31-40	0 (0)		7 (28)		0 (0)		0 (0)		0 (0)			6 (25)		0 (0)		0 (0)		0 (0)			6 (29)		0 (0)			0 (0)		
	41-50	1 (6)		11 (44)		0 (0)		0 (0)		1 (2)			6 (25)		0 (0)		0 (0)		2 (4)			9 (43)		0 (0)			0 (0)		
	51-60	1 (6)		6 (24)		0 (0)		1 (17)		8 (15)			7 (29)		0 (0)		0 (0)		14 (26)			3 (14)		0 (0)			0 (0)		
	61-70	7 (44)		0 (0)		2 (100)		0 (0)		21 (40)			3 (13)		1 (100)		0 (0)		22 (41)			2 (10)		2 (100)			0 (0)		
	71-80	6 (38)		0 (0)		0 (0)		5 (83)		15 (29)			0 (0)		0 (0)		2 (100)		14 (26)			0 (0)		0 (0)			1 (100)		
	>81	1 (6)		0 (0)		0 (0)		0 (0)		6 (12)			0 (0)		0 (0)		0 (0)		2 (4)			0 (0)		0 (0)			0 (0)		
**Race and ethnicity**																													
	Asian or Pacific Islander	0 (0)		1 (4)		0 (0)		0 (0)		2 (4)			0 (0)		0 (0)		0 (0)		1 (2)			1 (5)			0 (0)				0 (0)
	Hispanic or Latino	0 (0)		0 (0)		0 (0)		0 (0)		0 (0)			1 (4)		0 (0)		0 (0)		0 (0)			1 (5)		0 (0)			0 (0)		
	White	16 (100)		22 (88)		2 (100)		6 (100)		50 (96)			21 (87)		1 (100)		2 (100)		52 (96)			19 (91)		2 (100)			1 (100)		
	Other^e^	0 (0)		2 (8)		0 (0)		0 (0)		0 (0)			1 (4)		0 (0)		0 (0)		0 (0)			0 (0)		0 (0)			0 (0)		
	Prefer not to say	0 (0)		0 (0)		0 (0)		0 (0)		0 (0)			1 (4)		0 (0)		0 (0)		0 (0)			0 (0)		0 (0)			0 (0)		
**Education**																													
	Doctorate	1 (6)		1 (4)		0 (0)		2 (33)		5 (10)			1 (4)		0 (0)		2 (100)		5 (9)			1 (5)		0 (0)			1 (100)		
	Masters degree	5 (31)		1 (4)		0 (0)		2 (33)		14 (27)			2 (8)		0 (0)		0 (0)		16 (30)			2 (10)		0 (0)			0 (0)		
	Bachelors degree	5 (31)		16 (64)		0 (0)		0 (0)		12 (23)			11 (46)		0 (0)		0 (0)		19 (35)			11 (52)		0 (0)			0 (0)		
	High school degree or equivalent	1 (6)		6 (24)		0 (0)		2 (33)		10 (19)			6 (25)		1 (100)		0 (0)		6 (11)			6 (29)		1 (50)			0 (0)		
	Less than a high school diploma	1 (6)		0 (0)		2 (100)		0 (0)		4 (8)			1 (4)		0 (0)		0 (0)		2 (4)			0 (0)		1 (50)			0 (0)		
	Prefer not to say or other	3 (19)		1 (4)		0 (0)		0 (0)		7 (14)			3 (13)		0 (0)		0 (0)		6 (11)			1 (5)		0 (0)			0 (0)		
**Do you wear a wearable?**																													
	Yes	10 (63)		10 (40)		1 (50)		0 (0)		18 (35)			6 (25)		0 (0)		0 (0)		22 (41)			11 (52)		0 (0)			0 (0)		

^a^PD: Parkinson disease.

^b^MS: multiple sclerosis.

^c^COPD: chronic obstructive pulmonary disease.

^d^PFF: proximal femoral fracture.

^e^Additional race and ethnicity demographics include round 1 (2 mixed race participants, 1 White and Latino, and 1 Turkish or Northern Irish), round 2 (1 mixed race participant, Turkish or Northern Irish), and round 3 (1 Jewish participant).

### Round 1

Examples of the mapping process per long-term health condition are provided in Table 3. The experiences described by patients for each health condition are briefly summarized here.

For people with PD, scenarios and examples that related to the themes of *physical experience of walking*, *the context of the walking experience*, and *becoming aware of the personal walking experience* were frequently reported. They described how their symptoms affected their balance and noted that they felt they were not able to do as much because of their symptoms, which forced them to slow down. Participants also described how their walking changed depending on the time of day and location. This impacts where and how far they might be willing to go. They also described feeling more conscious of how they walk in terms of speed and quality to counter the changes to their balance. These experiences could be directly related to DMOs describing stride length, step duration, walking speed, and step count (Table 3), which reflect altered balance, speed of movement, and amount of activity.

People with MS frequently described scenarios and examples that related to the themes of *physical experience of walking*, *the context of the walking experience*, and *the walking experience as a link between individual’s activities and sense of self*. MS symptoms led people to describe how fatigue impacts their lives, influencing their ability to walk distances and to plan activities. They also spoke about how the symptoms of MS influenced their capacity to undertake activities, depending on how they felt on a daily basis. Finally, they described changes to their balance and perception of distance, which impacted their ability to walk smoothly. The DMOs that most frequently mapped to these themes were step count, stride length, walking speed, and step duration.

Limited data were collected from people with COPD or PFF. However, both groups focused on their physical symptoms, which appeared to link most closely to the amount of activity that they could do. People with COPD described breathlessness as an important symptom that led to struggling to walk and use stairs, among others, thus limiting the amount or duration of their physical activity. For people with COPD, these experiences mapped onto step count and number of walking bouts. People recovering from PFF described changes and adaptations since their fracture, including the need to walk slower, increased fatigue, and having to rest because of this, which mapped onto DMOs, such as walking speed and step count.

**Figure 2:**
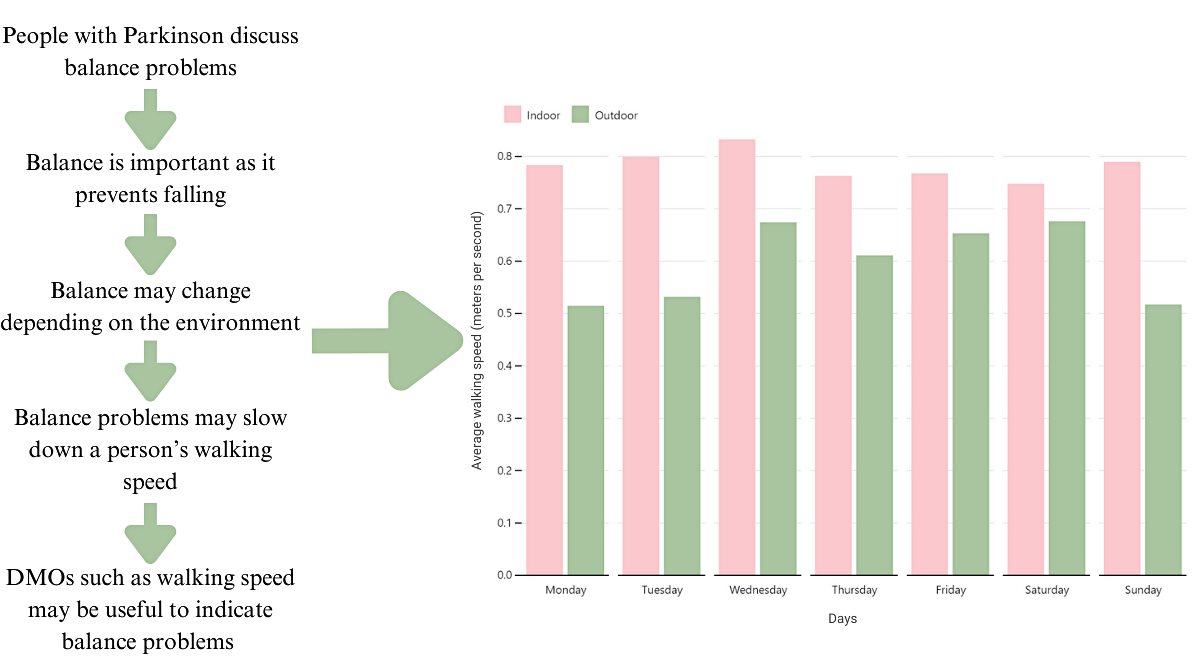
Representation of developing the visualizations of round 2 through the feedback from round 1. DMO: digital mobility outcome.

Following this step, the issues and concepts that were most often mentioned were listed by the health condition and used to identify the data required to develop visualizations in line with these needs. The most relevant DMOs and contexts were extracted from the Mobilise-D TVS and were drafted into visualizations to represent varying mobility impairments across the 4 health conditions; this process is presented in Figure 2 and Multimedia Appendix 3 [23].

**Table 3:** Examples of how the patient experiences were mapped to mobility themes and digital mobility outcomes (DMOs).

Example quotation			Mobility themes based on Delgado-Ortiz et al [3]		Mapped DMOs
**Patients with PD^a^**					
	“I walk slowly now so have to allow more time to get anywhere. My family and friends adapt their speed to me so no issues with interactions. I have had some issues in crowded football grounds or trains where I feel unsteady on my feet.” [White male participant with PD, aged 61-70 y]	The physical walking experience The social experience of walking Behavioral and attitudinal adaptations resulting from the walking experience Becoming aware of the personal walking experience		Stride length Walking speed Step duration	
	“Any way that I can improve my walking which is ok when outside when I concentrate hard on what I am doing, taking long steps and counting out loud. In the house I tend to stand still for ages before I can get my legs to move.” [White female participant with PD, aged 71-80 y]	The physical walking experience Behavioral and attitudinal adaptations resulting from the walking experience The context of the walking experience		Step count; WB^b^ duration Stride length Step duration	
	“Walking to the shops used to be 5 minutes up a hill but has become 10 to 15, my family are not impressed by the shuffle.” [White male participant with PD, aged 71-80 y]	The physical walking experience The social experience of walking The context of the walking experience Becoming aware of the personal walking experience		WB duration Walking speed	
**Patients with MS^c^**					
	“It tends to change depending on your energy, what you’ve done the day before and the weather. Today has been ok but my body feels tired.” [White female participant with MS, aged 31-40 y]	The physical walking experience The context of the walking experience		Walking speed Step count Number of WBs	
	“There is a goldilocks zone. Below minus 3 degrees or above 26 degrees then I walk like I’m drunk. I find this frustrating and left a walking group as a man teased me about it and kept trying to get others in the group to comment on my walking or smell my breath for alcohol. I only walk with my dog alone now.” [White female participant with MS, aged 51-60 y]	The physical walking experience The mental and emotional walking experience The social experience of walking The context of the walking experience Behavioral and attitudinal adaptations resulting from the walking experience		Stride length Walking speed Step duration	
	“Walking is normal today. depending on sleep and level of activity walking can become very slow and laboured. Also drop foot can occur and more balance/shoe scuffing issues.” [White male participant with MS, aged 41-50 y]	The physical walking experience The context of the walking experience.		Stride length Walking speed Step duration	
**Patients with COPD^d^**					
	“Yes my walking and climbing stairs impact my breathing on most days I struggle going up stairs I try to keep myself downstairs once I get up.” [White female participant with COPD, aged 61-70 y]	The physical walking experience The context of the walking experience Behavioral and attitudinal adaptations resulting from the walking experience		Step count	
	“If I do too much I get very breathless and have to rest I also plan what I’m going to do to help my breathing.” [White female participant with COPD, aged 61-70 y]	The physical walking experience Behavioral and attitudinal adaptations resulting from the walking experience		Step count Number of WBs	
**Patients with PFF^e^**					
	“My walking has a great influence for my daily activities—I have only few social interactions with friends and no hobbies.” [White female participant with PFF, aged 71-80 y]	The social experience of walking Behavioral and attitudinal adaptations resulting from the walking experience The walking experience as a link between an individual’s activities and sense of self		Step count	
	“I don’t walk as fast anymore, I don’t jog or cycle anymore. I go to physiotherapy regularly. My relatives rarely talk about my fracture.” [White male participant with PFF, aged 71-80 y]	The physical walking experience The social experience of walking Behavioral and attitudinal adaptations resulting from the walking experience		Step count Walking speed	

**^a^**PD: Parkinson disease.

^b^WB: walking bout.

**^c^**MS: multiple sclerosis.

**^d^**COPD: chronic obstructive pulmonary disease.

**^e^**PFF: proximal femoral fracture.

### Round 2

**Figure 3:**
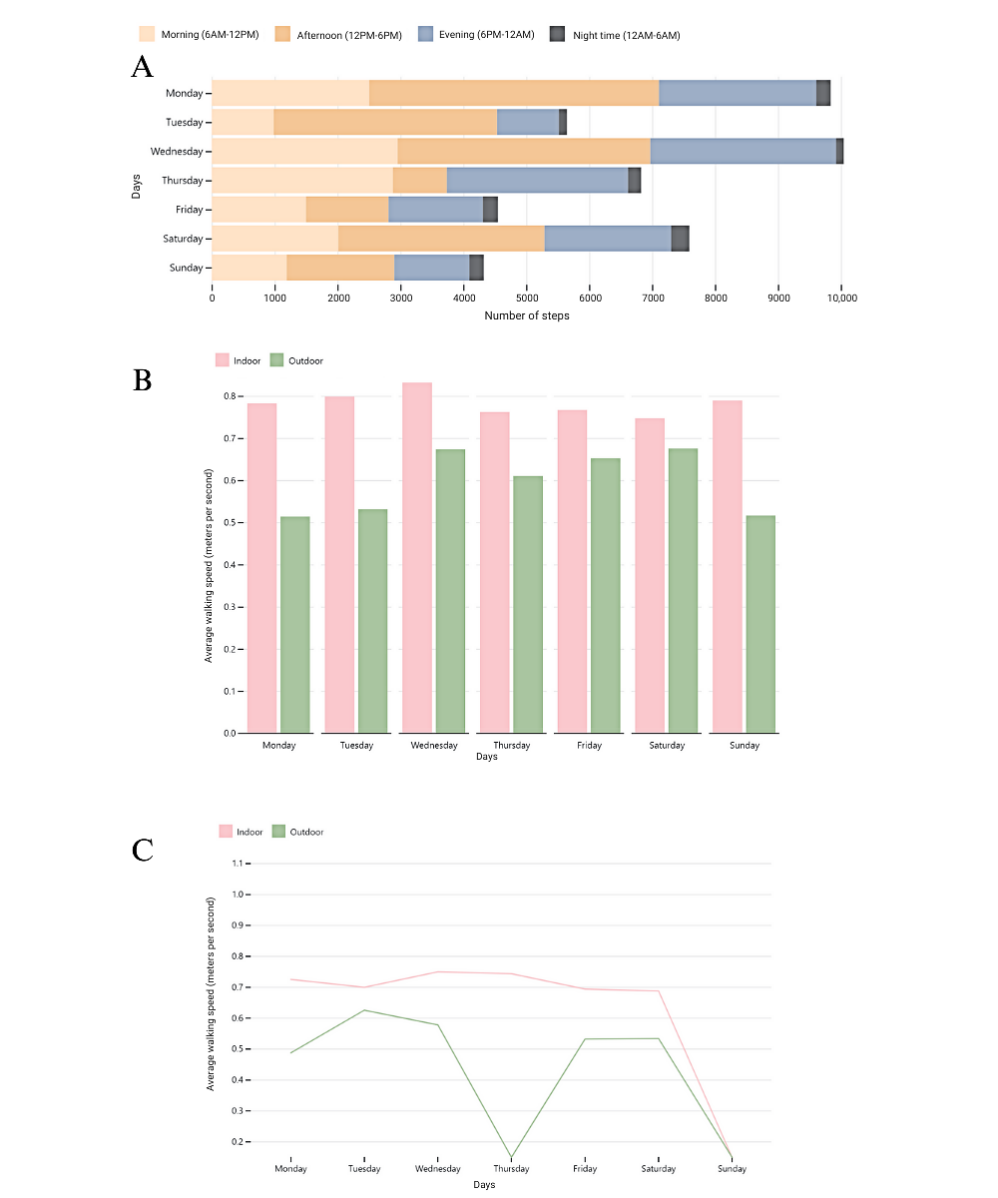
Examples of draft visualizations presented to participants in round 2. A: Horizontal stacked bar chart showing number of steps across a week and through each day to indicate fatigue B: Bar chart showing average walking speed across a week both indoors and outdoors to indicate fatigue. C: Line chart showing average walking speed across a week both indoors and outdoors to indicate balance.

Researchers developed and presented 15 visualizations and justifications to participants based on the feedback from round 1. Every long-term health condition was presented with 2 visualizations of fatigue, PD was presented with 2 visualizations on balance, MS was presented with 3 visualizations on doing less, and PFF was presented with 2 visualizations on slowing down (Figure 3 gives the examples). Participants then rated the usefulness and ease of understanding of each visualization. The consensus was reached for the ease of understanding in people with PD for 2 graphs, namely the bar chart showing the indoor versus outdoor data for both balance and fatigue.

The qualitative responses to the draft visualizations were generally positive (summary in Table 4). Participants reported that they were able to understand the visualizations, and some were able to interpret what these visualizations represented. For some, particularly those with PD, more data and variables were deemed necessary to allow a fuller picture of the data being presented. Specifically, people with PD highlighted a desire for additional, contextual information and patient-reported outcome measures to accompany the visualizations. However, there were individuals in each long-term health condition that found the visualizations difficult to understand.

**Figure 4:**
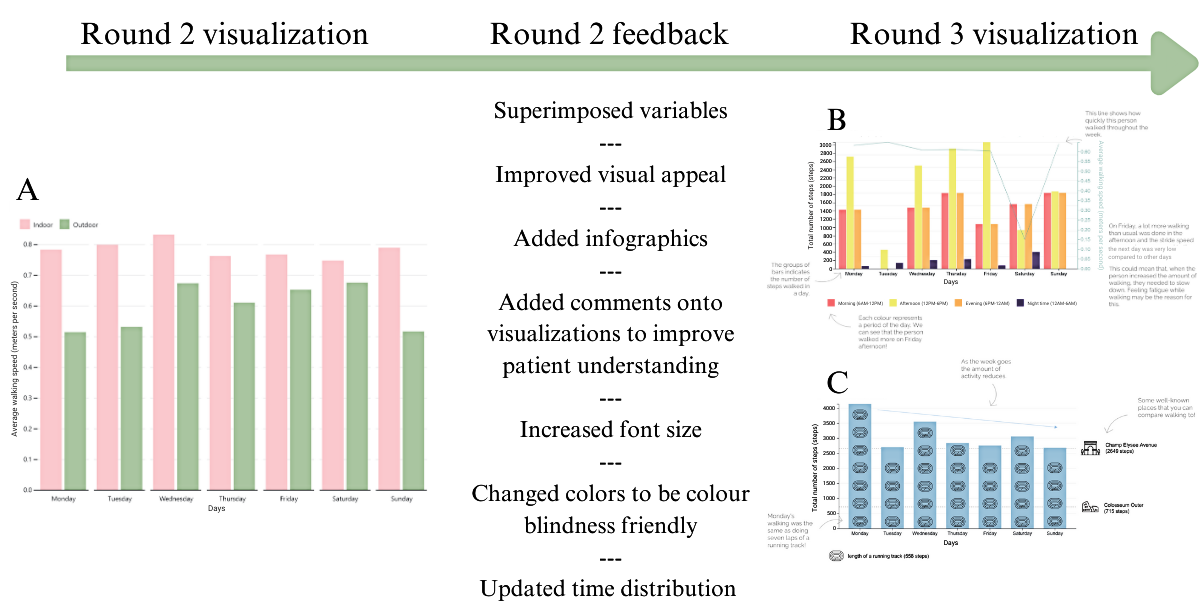
The process of refining the draft visualizations for round 3 based on the feedback from round 2. A: Bar chart showing average walking speed across a week both indoors and outdoors to indicate balance. B: Bar chart showing number of steps across a week and throughout each day superimposed by a line chart of average walking speed to indicate fatigue. C: Bar chart showing number of steps across a week to indicate fatigue.

A range of feedback was provided that touched on different aspects of the data visualizations and their method of presentation (Table 5 gives the summary). This was used to improve the visualizations for round 3 (Multimedia Appendix 1). Feedback such as superimposing variables and improving the clarity, accessibility, and visuals of the visualizations was implemented, as seen in Figure 4. Some feedback could not be implemented due to software limitations, such as adding an interactive element to switch between meters and feet.

**Table 4:** A summary of the insights gained from participants during round 2.

Themes	Examples of accompanying quotes
Ability to understand the visualization	“Easy to understand, maybe brighter colours would make it easier to read. I like that it spans across a week as MS can change day to day.” [White female participant with MS^a^ aged 18-30 y] “Easy to read the information and digest it” [White female participant with MS aged 41-50 y]
Ability to interpret the data	“Suggests that the subject moves around more confidently indoors than out.” [White male participant with PD^b^ aged 71-80 y] “It looks like after a few busy days fatigue sets in.” [Northern Irish and Turkish female participant with MS aged 41-50 y]
Desire for additional information	“I think the information could be very useful if there was a way to link to notes on how you were feeling on a given day to see what may have impacted your stats.” [White female participant with PD aged 51-60 y] “OK, this graph also shows indoor walking speeds are consistently higher than outdoor, but it also shows a varying degree of difference on different days, implying another factor at work—maybe the distance walked outside on different days?” [White male participant with PD aged 61-70 y] “It is useful to be able to see the difference between outdoor and indoor walking. It might be useful to record whether or not a mobility aid is being used and the type of surface being walked on.” [White female participant with PD aged 61-70 y]

^a^MS: multiple sclerosis.

^b^PD: Parkinson disease.

**Table 5:** A summary of suggested changes from participants during round 2 and the changes that were made in round 3 following these suggestions.

Issue raised	Examples of accompanying quotes	Change made
Superimposed variables	“I think that there has to be a better way to show stride cadence. Speed of step is one important factor, but length of stride is another. So, stepping out to say marching music with a quick march tempo helps to increase pace but what slows people with PD down is often that they take small steps to overcome uneven ground, and this also slows them down.” [White female participant with PD^a^ aged 61-70 y] “I would like to see the step length line chart superimposed over this bar chart. Small steps mean more steps over the same distance. As Alexander says ‘simples.’” [White female participant with PD aged 71-80 y]	Added more variables per graph to allow for deeper insight into the data shown.
Difficulty with the visualizations	“Too complicated, too confusing.” [White female participant with COPD^b^ aged 61-70 y] “Boring, too academic, too SPSS-like [data analysis software].” [White male participant with PFF^c^ aged 71-80 y] “My response was, oh, boring. More of the same! For the ordinary everyday person having to study these graphs.” [White female participant with PD aged >81y]	Made the graphs more visually appealing and added graphics to enhance the visualization.
Interactivity of graphs	“Assuming that it’s plotting the same data, then providing the user with the choice of how the data is displayed would be the most user-friendly approach.” [White female participant with PD aged 61-70 y]	Unable to integrate this feedback into round 3.
Improve clarity for the justifications of each graph	“So many other issues slow walking, all would need to be taken into account, such as gait, balance issues, foot drop, pain, as well as fatigue.” [White female participant with MS^d^ aged 51-60 y]	Made the link between participant experience and the mapping onto the DMO^e^ and context clearer.
Improve descriptions	“There is no info on how this data has been collected. Does the graph represent one person or is it the average of various persons?” [White female participant with PD aged 71-80 y] “This graph I found more informative. Is it possible to break it down to age, how long diagnose?” [White female participant with PD aged 51-60 y]	Made it clearer that these are single person’s data and are drafted as an example.
Improve accessibility	“The beige colours seem too close in hue.” [White female participant with MS aged 51-60 y] “Easy to understand perhaps colours could be brighter.” [White female participant with PD aged 71-80 y] “Unable to see colours as colour-blind to parts of spectrum, which is common in MS optic neuritis.” [White female participant with MS aged 51-60 y] “Very Faint” and “Too Pale” [White male participant with PD aged 71-80 y]	Increased font size and made lines on the line chart bolder. Changed to brighter colors that are more accessible for people with color blindness.
Change time distributions	“The information is good, I like the idea of being able to compare times of day, however I think the nighttime slot is too short and should be at least 10 hours. The evening slot is too long and shouldn’t go past 10pm. A better distribution would be Morning 7am-12pm; afternoon 12pm-5pm; evening 5pm-10pm, night 10pm to 7am.” [White female participant with PD aged 51-60 y]	Updated the times on event visualizations.

^a^PD: Parkinson disease.

^b^COPD: chronic obstructive pulmonary disease.

^c^PFF: proximal femoral fracture.

^d^MS: multiple sclerosis.

^e^DMO: digital mobility outcome.

### Round 3

**Figure 5:**
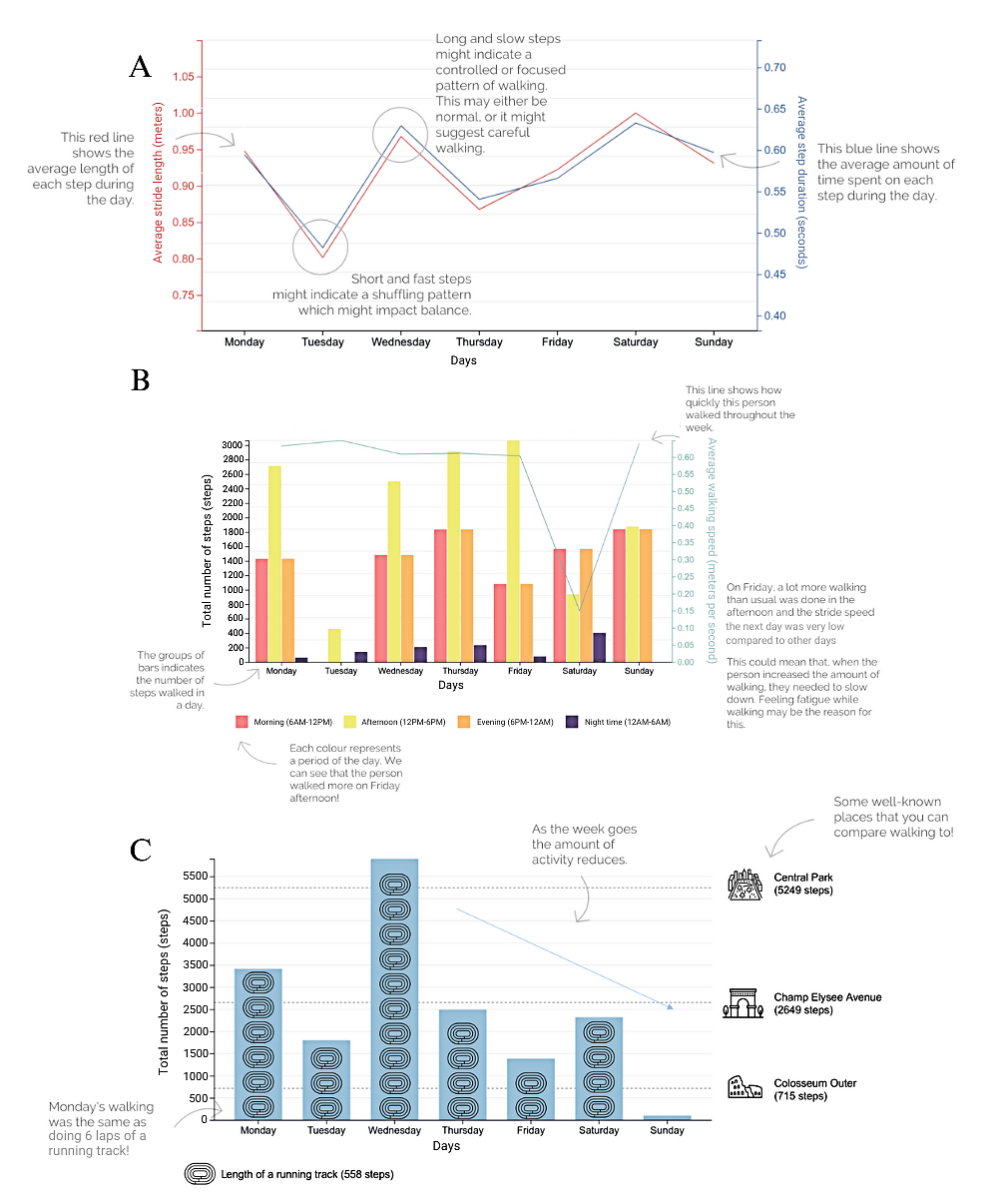
Examples of draft visualizations presented to participants in round 3. A: Line chart showing average stride length and average step duration across a week to indicate balance. B: Bar chart showing number of steps across a week and throughout each day superimposed by a line chart of average walking speed to indicate fatigue. C: Bar chart showing number of steps across a week to indicate fatigue.

Researchers developed and presented 8 visualizations and justifications to participants based on feedback from round 2. Every long-term health condition was presented with 2 visualizations of fatigue, PD was presented with 2 visualizations on balance, MS was presented with 3 visualizations on doing less, and PFF was presented with 2 visualizations on slowing down (Figure 5 gives the examples). Patients rated the usefulness and ease of understanding of each visualization. No consensus was reached during round 3 for any visualization.

Qualitative responses to the draft visualizations were generally positive (summary provided in Table 6). Participants reported that they generally understood the visualizations and appreciated the comments surrounding the visualizations that explained the graph and how to interpret the outcomes. Participants found the differentiation between environment and time of day included in the visualizations to be important, as this influences the presentation of their symptoms.

A range of feedback was provided throughout the comments touching on different aspects of the data visualizations and how they were presented (aggregated summary in Table 7). This feedback could not be used within this study but serves as a recommendation for future studies wishing to visualize mobility data. The feedback mainly surrounded accessibility and explanations, as well as the need for additional data to fully interpret the graphs. Interestingly, this round provided contradictory feedback between participants, with some appreciating the infographic visuals while others found them irritating. This indicates the need for personalization of data visualizations.

**Table 6:** A summary of the insights from participants gained during round 3.

Themes	Examples of accompanying quotes
Appreciated the comments to explain the graphs	“Looks good and explained well” [White female participant with COPD^a^ aged 61-70 y] “I find this graph harder to follow than the previous one. Without the explanatory notes, not sure I’d have grasped the logic behind the walking speed trend line.” [White male participant with MS^b^ aged 41-50 y]
Easily understood the graphs	“Simple and clear graphics made graph easy to understand. The purpose of each data item shown was clearly described. I would find this graph very helpful.” [White male participant with PD^c^ aged 51-60 y] “Easy to understand, colours differentiate it quite well.” [Hispanic female participant with MS aged 18-30 y]
Time of day and location are important to understand symptoms	“Personally, my stride length varies not just with location (indoors/outdoors) but also where I am in the drug cycle...So it would be useful to not only plot the steps per day but throughout the day.” [White female participant with PD aged 61-70 y] “Round the house I take short quick steps, sometimes with festination and stumbles. At Sainsburys [a supermarket] with a trolley I am much more normal in my stride length and speed” [White female participant with PD aged 71-80 y]

^a^COPD: chronic obstructive pulmonary disease.

^b^MS: multiple sclerosis.

^c^PD: Parkinson disease.

**Table 7:** A summary of the suggested changes from participants during round 3.

Issue raised	Examples of accompanying quotes
Graphs are small	“The graph is small and difficult to read therefore difficult to interpret.” [White female participant with PD^a^ aged 61-70 y]
Explanations are too faint	“I’ve had to zoom in to read the comments on all the graphs—could they not be black, rather than grey?” [White female participant with PD aged 51-60 y] “The explanations of the key features of the graph would be easier to read if in a larger font size and bolder type.” [White female participant with PD aged 51-60 y]
More contextual information is needed	“It would be useful to know if a walking aid was used.” [White female participant with MS^b^ aged 41-50 y] “I don’t see the purpose of monitoring strides etc unless you show a graph as to how and why the people involved changed their stride length. Parkinson’s varies daily, illness affects symptoms, etc.” [White female participant with PD aged 61-70 y] “There doesn’t seem to be any way of showing e.g. gradient or quality of surface that you are walking on which limits the usefulness of this graph.” [White female participant with PD aged 61-70 y]
Debate surrounding the visuals used in some graphs	“I think this is very easy to understand. I like visual graphs rather than data.” [White female participant with MS aged 41-50 y] “I am not sure I like the running tracks.” [White female participant with MS aged 61-70 y] “This graph seems to have little point beyond the step count. the graphics (colosseum etc) are unnecessary and, for me, irritating...adding CeeBeeBee [a children’s TV channel in the UK] style graphics doesn’t add anything useful.” [White male participant with PD aged 71-80 y] “I like this graph. Easy to understand and I really like the Running Track and famous places comparisons for distances.” [White male participant with PD aged 51-60 y] “I love the idea of the equivalent places, but could this be more localised/personalised?” [White female participant with PD aged 51-60 y]
The link between DMO^c^ and symptoms is unclear	“My walking speed can be quite fast even though I’m experiencing fatigue, because of foot drop.” [Hispanic female participant with MS aged 18-30 y] “I think that this doesn’t help express fatigue.” [White female participant with MS aged 41-50 y] “People with Parkinson’s sometimes shuffle and take small steps, so this will help to understand if or how often they occur.” [Asian or Pacific Islander female participant with PD aged 61-70 y]
Questions on the usefulness of the graphs	“It describes the situation but I’m not sure how I would utilise the information.” [White female participant with PD aged 61-70 y] “Not clear what use this graph for” [White male participant with PD aged 61-70 y] “Although the graph is easy to read and gives a clear visual representation of the number of steps taken, is this information really useful? If you are tired clearly you will want to walk less.” [White female participant with PD aged 51-60 y]
The need for a healthy control’s average	“Might a ‘normal’ comparator be useful? adjusted for gender and age. i.e. are my steps tiny compared to other women of my age??” [White female participant with PD aged 71-80 y]

^a^PD: Parkinson disease.

^b^MS: multiple sclerosis.

^c^DMO: digital mobility outcome.

## Discussion

### Principal Findings

This study explored patient preferences on how mobility data derived from wearable devices is visualized. Through the multistage cocreation of visualizations with patients as experts, we embedded the voice of patients throughout the research cycle so that the visualization recommendations would be meaningful to them. While these early insights cannot claim a consensus of patient preferences on mobility data visualizations, the study provides important learnings and recommendations for future mobility visualizations.

Participants were generally able to understand the visualizations and interpret what the data may indicate. The next step would be advancing and personalizing the data visualizations to empower patients to gain actionable insights from their inferences. Feedback consistently requested additional data to be presented to enhance the visualizations; this was achieved through embedding indoor versus outdoor categories in certain visualizations as well as superimposing DMOs on some visualizations in round 3. Participants appreciated this extra detail during round 3; however, this was not sufficient in terms of the information they felt was important to help them apply learnings or actions into their daily lives.

Contextual information, such as how the person felt, when medication was taken, and whether they used a walking aid, would be important when interpreting the visualizations. Previous studies have explored the use of calendars to present medication data over time using a similar approach; it would be possible to note when medication changes or interventional changes occur by indicating them with icons [19,21]. Alternatively, exploring the granularity of data may be helpful in addressing these comments; for example, exploring when medication was taken throughout the day may lend itself better to visualizing a single day’s data with icons added when medication was taken to identify any trends related to medication [18,35]. Previous patient-focused visualization studies within literature also discuss the importance of context and additional factors in determining the meaningfulness of a visualization; hence, it is important for these contexts to be embedded into visualizations [12,15,36]. Typical consumer wearable devices display a myriad of health data, such as number of steps and calories burned; however, these visualizations are often oversimplified and, consequently, do not demonstrate the relationship between multiple factors or acknowledge the presence of external, contextual factors [37]. It is important that researchers do not default to the basic bar charts of consumer wearable devices and instead recognize and highlight the intertwined nature of health, environment, and activity, particularly in those with health conditions whereby this additional information is vital.

Some participants found it difficult to interpret the information from the visualizations and apply it to themselves. This is potentially due to insufficient justifications, descriptions, and accessibility of the visualizations [12]. This indicates the importance of working alongside patient advisors to understand the lived experience of long-term health conditions and develop justifications for what and why the data are displayed [32,38]. A major barrier to understanding for participants was the rationale for why certain DMOs had been chosen to represent a symptom. This indicates the need for future qualitative studies to be conducted to map the patient experience to each DMO. Research to understand the link between patient experience and outcome measures is limited but will be essential for understanding the importance of where DMOs fit into the patient experience. Being able to use visualizations to make sense of the “chaos of lived experience” will be key to encouraging the use of wearable devices within health care [28]. While this mapping process was piloted within round 1 of this study, a larger and more in-depth mapping process is needed to bridge the gap of DMO meaningfulness to patients. Already, the benefits of this mapping process have been seen in other areas of digital health technologies, with additional studies currently ongoing [39-41].

An important barrier to interpreting the visualization was a lack of personal connection with the data. As the data presented to participants was not their own, they found it difficult to understand what was being visualized, as there was no context for what the data implied. This was a recurring theme throughout the qualitative analysis and something that should be addressed in future studies by personalizing visualizations to patients’ own data to address these barriers. Visualizing personal data may empower patients to make actionable insights that reflect their own lived experiences, which makes strides toward the potential for personalized health care [42]. This may also address the lack of consensus found in this study, as participants had no familiarity with the data presented, and this may have contributed to poorer understanding and usefulness of the data.

Patients were able to read and interpret bar charts, but their ability to interpret DMOs, such as stride length and walking speed, was hindered due to a lack of understanding of a healthy control’s “normal.” This is a common theme within clinical data visualizations. Providing a “normal” value may be helpful when goal setting using visualizations; however, issues arise regarding how that “normal” value is set and ensuring it reflects the age, gender, and other characteristics of the patient [21]. Other studies demonstrate that the use of visual cues, such as emojis, and user-friendly visual analogies can improve the understanding of the visualization [36,43]. The use of visual cues throughout data visualization should be of particular importance in future work with difficult-to-visualize outcomes. During round 3 we included comments and additional visual cues to better explain the visualization. The feedback implied that this was well received, yet there is scope for further enhancing these visual cues and comments. For example, the ability to relate to famous monuments as a visual cue was very individualized and should be personalized based upon participant location for future visualizations. Moreover, the accessibility of the graph is important. Due to software limitations, the graphs presented were small and difficult to read for some participants; this is something to be mindful of in future studies.

Future studies should explore alternative visualization strategies. Here, data were presented using graphs to display changes during a week and to represent the dynamic nature of patient experiences. However, certain DMOs may be better conveyed through other visual methods. For example, some studies have used motion capture and videos to visualize gait, which may be an interesting avenue for future mobility-related visualization studies [44,45]. Furthermore, for data to be relevant to a person’s immediate context, future work should focus on designing a tool that can provide real-time feedback that supports people’s understanding of their condition [27].

### Limitations

The COPD and PFF health conditions had a lower representation and did not meet the recommended number of participants for Delphi protocols (10 to 15 participants); this limits the understanding we have of how these groups prefer their data to be visualized [31]. This was due to our recruitment methods not being successful for these health conditions. While results indicate that DMOs and justifications should be personalized based on the symptoms and experience of a long-term health condition, the sample size was not adequate to explore any differences in visualization preferences between groups. Further research is necessary to fully understand any nuances that may arise from these long-term health conditions.

The sample of this study was not representative; participants were largely educated from Western countries (primarily the United Kingdom) and around a third of participants used a wearable device in their daily lives. Due to ethical requests, participant demographics were only collected after participants were recruited; it was not possible to retrospectively recruit and address this disparity. In addition, by nature of a web-based survey, it is likely that the digitally excluded were not well represented in this study. It is pivotal that research includes and represents the experiences of underserved communities to improve the generalizability of findings [46,47]. Previous data visualization studies have looked at preferences in people with lower educational attainment; this highlights the importance of diversity within data visualization samples, as preferences may vary between groups [48]. For the integration of wearable devices into health care, it will be paramount to understand how best we can visualize DMO data to ensure a wide range of demographics can access and understand the data provided. To address this, future research should aim to provide a more representative sample [49].

It is essential that future studies are conducted with clinicians and those involved in the care of patients. With the rise of commercial wearable devices, data are being presented to clinicians by patients and being dismissed, as there is a lack of understanding on the clinician’s side for how to interpret and act on the data [21]. It is imperative that the people involved in a person’s care (both the patient and care team) can extract meaningful data from wearable devices. It may be that different forms of visualization may be helpful for clinicians compared to patients; potentially clinicians will be more interested in longitudinal changes, such as baseline to 6 months, than patients. Already studies have explored this for clinic-based sensor data, and this should be explored more widely using real-world data to capture the nuances of mobility [50].

### Conclusions

The use of remote mobility monitoring devices is predicted to increase. Hence, how to visualize this data in an understandable and meaningful way to patient populations is of key importance. This study indicates that visualizing these data is possible and can be done in a way that patients understand. While this study did not draft any visualizations that had a strong consensus of understanding and ease of use, we were able to provide several points of recommendation for future visualizations and studies. Particularly, we recommend that future studies explore individualized visualizations, whereby the participant’s own data are visualized both individually and in the context of their condition to better enhance understanding.

### Recommendations

Despite the exploratory nature of this work, we have been able to develop several initial recommendations for future visualizations in tandem with existing principles that are applicable both for the visualization of mobility data as well as the visualization of health data in general, which include the following:

- Ensure readability—visualizations should be large, easy to read, and contain sufficient color contrasts [51,52].
- Be aware of potential visual impairments within individuals and conditions, for example, contrast sensitivity in MS due to optic neuritis, as well as individual additional needs that must be catered to [53,54].
- Be aware of the need for additional information. Patients understand the variability of their condition best; hence, providing the ability to dynamically interact and insert additional information to explain this variation would be greatly beneficial [12,15,36].
- Think beyond the basics of consumer device visualizations. Experiment with different visualization techniques to enhance patient understanding [19,21,43].
- Ensure a strong rationale. Engage with patients and embed public and patient involvement within the project to ensure meaningful outcomes for patients [38,55].
- Use individualized visualizations. Personalize visualizations to the data of each participant; while not done in this study, participants indicate this as the next step [42].

## Appendix

Multimedia Appendix 1 Examples of text and visualizations presented to participants.

Multimedia Appendix 2 Coding framework, based on Delgado-Ortiz et al [3] and coding framework of health condition–specific walking elements (foot drop and freezing, among others).

Multimedia Appendix 3 Technical validation study data requirements.

## Abbreviations

COPD: chronic obstructive pulmonary disease
DMO: digital mobility outcome
MS: multiple sclerosis
PD: Parkinson disease
PFF: proximal femoral fracture
PPAG: Public and Patient Advisory Group
TVS: technical validation study

## Data Availability

The data collected and analyzed within this study are available upon request from the corresponding author.
