# Supplementary material for: Cocreating the Visualization of Digital Mobility Outcomes: Delphi-Type Process With Patients
Source: JMIR Form Res. 2025 May 9;9:e68782. doi: 10.2196/68782 (PMC12102624; doi:10.2196/68782)
Supplement: Multimedia Appendix 1 [file formative_v9i1e68782_app1.docx]

| **Round 1 - Example** |
| --- |
| When we talk about walking, we mean any activity that requires you to walk, including practical and every day activities (e.g. going to the shop, going upstairs, going for a walk with friends, housework etc.). Although it includes exercise based walking, it includes more than simply that.  We have provided a brief example on the next page of a person with a chronic condition that impacts their walking and what they experienced as a result of their condition. We do not wish for you to replicate this example, but we wanted to provide a general idea of what way others have described this impact before.  Much more detail can be added to each question should you like, but essentially we are asking you to describe your experiences, how they change, and what information you would like to know about yourself if possible.  “Today the main impact is a certain loss of balance and stability. My walking range is now about 1km but I have to concentrate or I walk like I am drunk. At times, I also misjudge distances and occasionally bump into people in the street. Standing still for any period of time is difficult as I soon feel like I am swaying. Lately, I have had episodes of freezing which nearly resulted in falls."  Please describe how your walking is today?  For example, are you aware of any physical symptoms that are impacting it? Does your walking affect your feelings or emotions today?  Is your walking today normal for you or does it tend to change?  If you tend to experience changes to your walking day to day, can you give us examples of these changes?  In general, how does your walking impact your daily activities (i.e. hobbies, work, basic tasks) and your social interactions (i.e. with family, colleagues, friends, strangers etc.)?  Would you like to receive information about your walking in relation to your condition? Why/why not?  Examples of information might be if changes in your walking are related to changes in your condition. Whether you are able to walk further or less than 6 months ago, or whether your balance has changed, etc.  If you would like to receive information about your walking, what kind of information would you like to know? |
| **Round 2 – PFF Example** |
| Based on this analysis we identified the following patterns within people with a hip fracture:  People with a hip fracture spoke about doing less and feeling tired. They also mentioned that they slowed their walking. This can be influenced by whether they are indoors or outdoors.  As a result of this, we developed a number of draft visualisations to represent the type of information that may be important to you. Each of these graphs relate to an individual person's data and is representative of what each individual person may receive. We would now like your opinion on whether these visualisations are useful to you or not.  These graphs demonstrate information about the speed of our walking. People report that recovery from hip fracture can impact how quickly they feel that they can walk. This may be due to confidence after the fracture, or it may depend on the stage of recovery. The walking parameters that we study in Mobilise-D, such as stride speed can be related to how quickly we walk. This graph has been designed to visualise a person's stride speed per day, at different periods in a day.  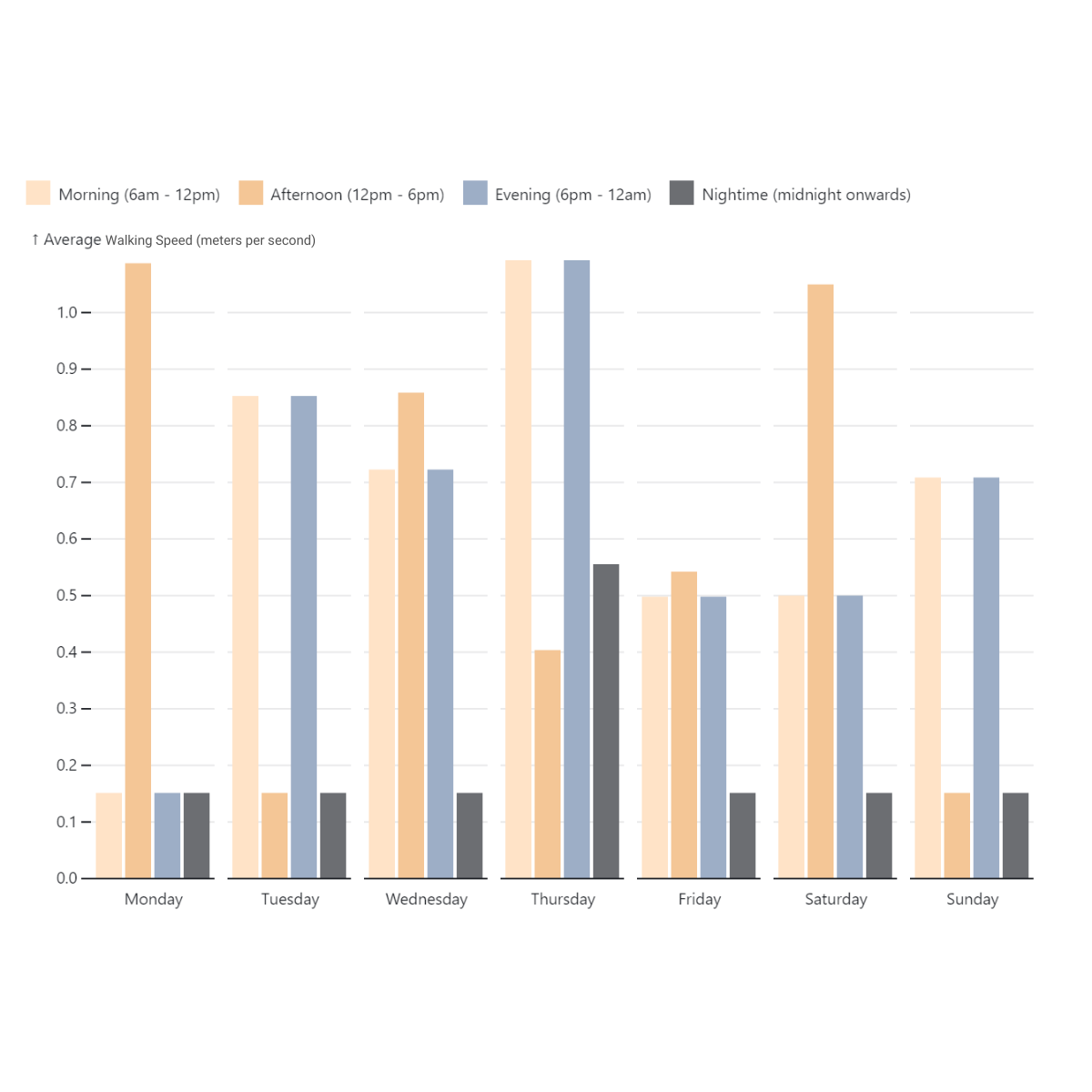 |
| **Round 2 – MS Example** |
| Based on this analysis we identified the following patterns within people with MS:  People with MS mentioned that they experience fatigue, find themselves doing less, weakness in their legs and having to take frequent breaks. They notice these experiences more between different days, and the weather can be an important feature. Whether they are indoors or outside can also be impactful.  As a result of this, we developed a number of draft visualisations to represent the type of information that may be important to you. Each of these graphs relate to an individual person's data and is representative of what each individual person may receive. We would now like your opinion on whether these visualisations are useful to you or not.  These graphs demonstrate information about fatigue. Fatigue is linked to mobility because if we feel fatigued, we may struggle to complete the activities that we wish to during the day, with the same energy or vigour as we might like.  People suggested that fatigue can occur quite quickly and that it can be a struggle to complete tasks as we might like. The walking parameters that we study in Mobilise-D, such as stride speed, can be related to fatigue, as if you feel fatigued you may expect to slow down the pace of your walking. This graph has been designed to visualise the number of steps per day, both indoors and outdoors.  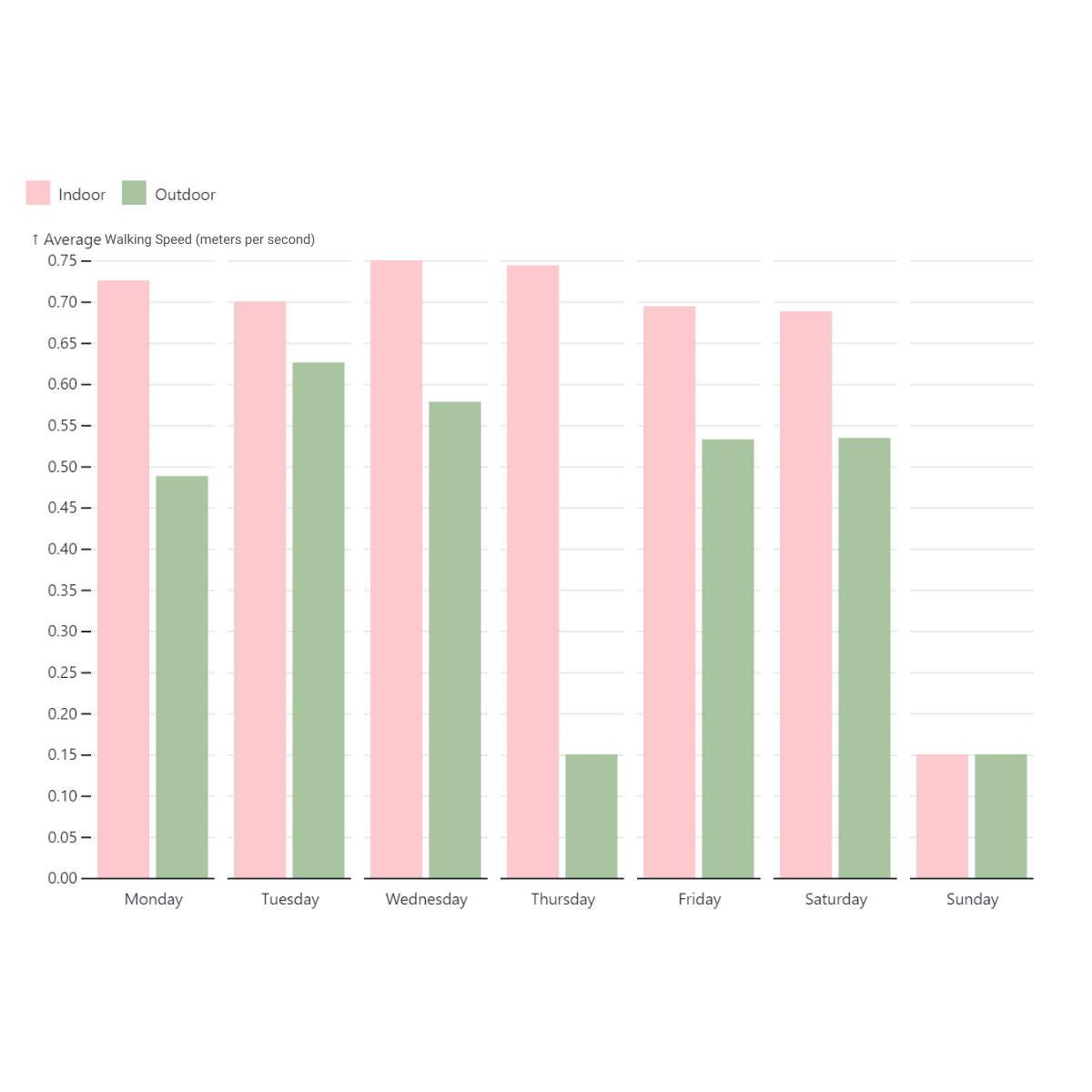 |
| **Round 3 – COPD Example** |
| These visualisations have been created using the data from a wearable sensor. They represent the data of a single individual’s walking. These visualisations are not meant to represent everyone with Chronic Obstructive Pulmonary Disease, but instead serve as a means for exploring preferences in the visualisation of mobility data. They are designed to be used as an example of what information might be provided to individuals and how, in order to understand how best to present information over time.  This graph demonstrates information about fatigue. Fatigue is linked to mobility because if we feel fatigued, we may struggle to complete the activities that we wish to during the day with the same energy or vigour as we might like. People suggested that changes in COPD symptoms such as shortness of breath can impact how a person feels from day to day. The walking parameters that we study with wearable sensors, such as the number of steps can be related to what we do in a day.  If changes to symptoms occur, you may take more or less steps, depending on how you feel. This graph has been designed to visualise the number of steps per day.  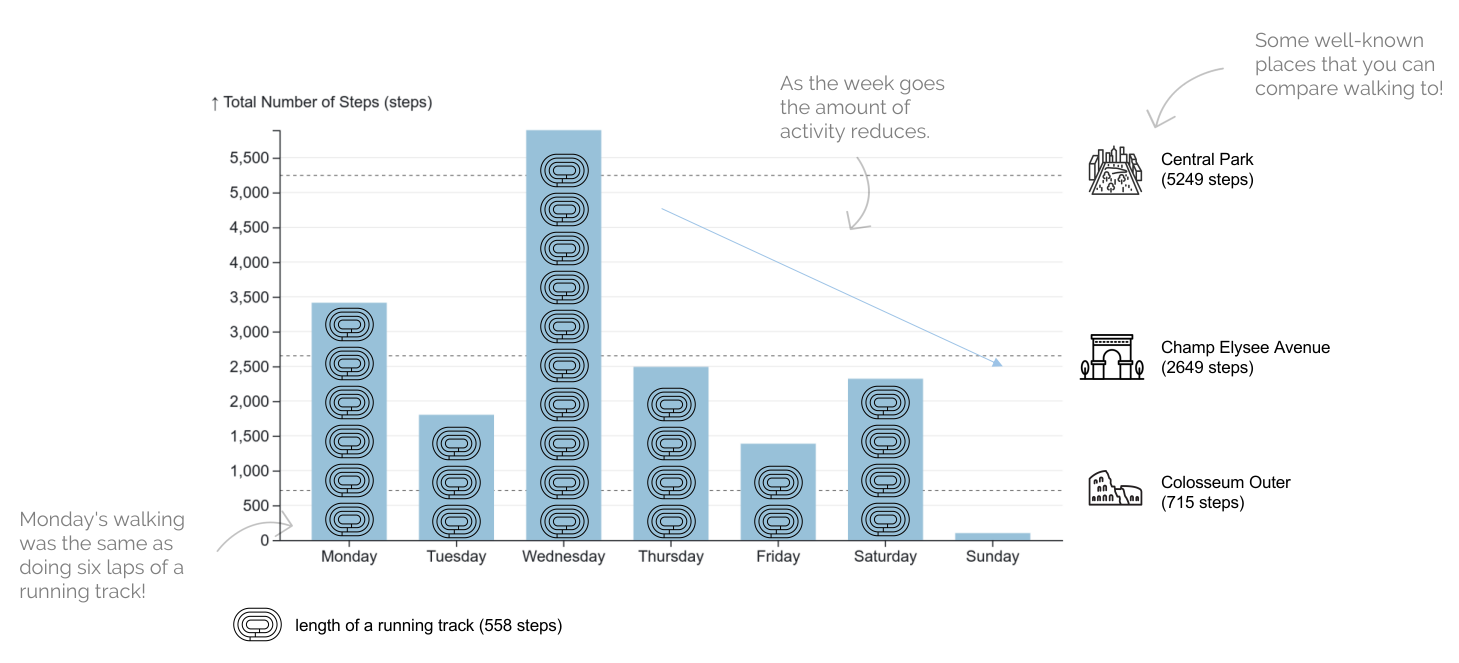 |
| **Round 3 – PD Example** |
| These visualisations have been created using the data from a wearable sensor. They represent the data of a single individual’s walking. These visualisations are not meant to represent everyone with Parkinson’s Disease, but instead serve as a means for exploring preferences in the visualisation of mobility data. They are designed to be used as an example of what information might be provided to individuals and how, in order to understand how best to present information over time.  This graph has been designed to demonstrate information about fatigue. Fatigue is linked to mobility because if we feel fatigued, we may struggle to complete the activities that we wish to during the day. People often note that doing less is a key impact of their Parkinson’s disease on their day. The walking parameters that we study with wearable sensors, such as the number of steps and stride length, can be related to fatigue.  If you feel fatigued, you may expect to take less steps and you may find that your gait changes. This graph has been designed to visualise the number of steps per day, both indoors and outdoors, as well as showing the average stride length across days.  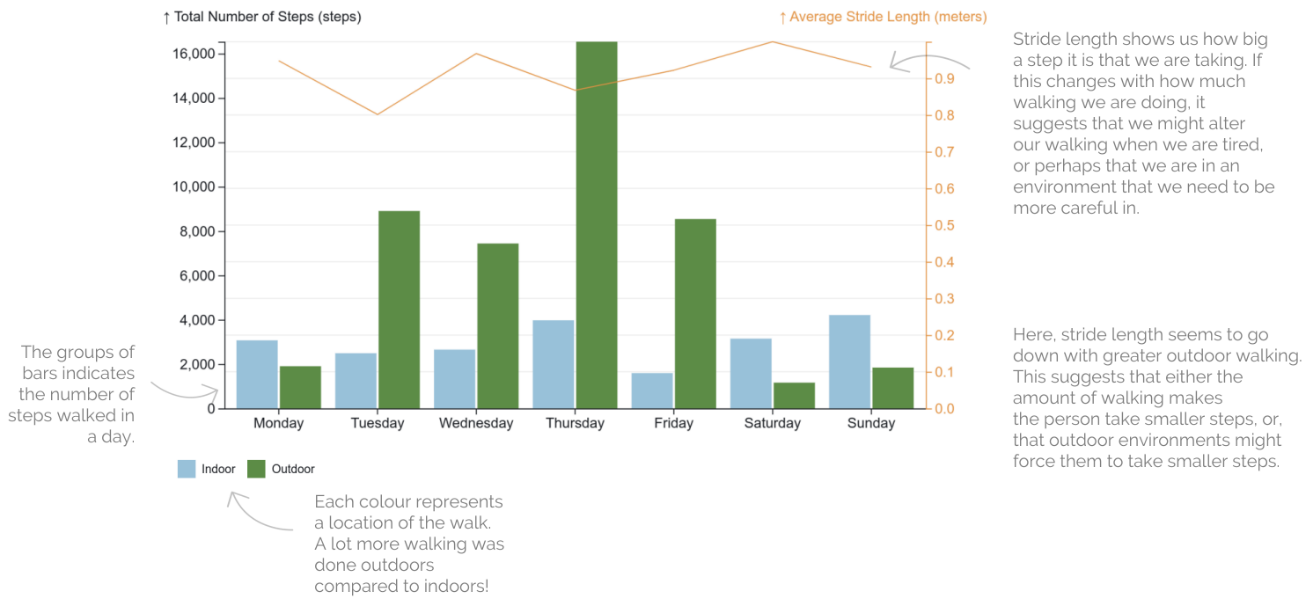 |
