# Supplementary material for: Cocreating the Visualization of Digital Mobility Outcomes: Delphi-Type Process With Patients
Source: JMIR Form Res. 2025 May 9;9:e68782. doi: 10.2196/68782 (PMC12102624; doi:10.2196/68782)
Supplement: Multimedia Appendix 2 [file formative_v9i1e68782_app2.docx]

| **Coding domain** | **Definition** |
| --- | --- |
| **Mobility framework theme** | |
| Becoming aware of the personal walking experience | In the presence of signs and symptoms of mobility-impairing health conditions, walking deteriorates and begins to require conscious effort, resulting in an awareness of walking which is not present when walking is unimpaired. |
| The walking experience as a link between individual’s activities and sense of self | Walking is symbolic of independence, control, normalcy and well-being because it governs one’s ability to engage in activities safely. When walking impairments affect activities associated with domestic and social roles, they indirectly challenge individuals’ sense of self. |
| The physical walking experience | Individuals consistently described a physical experience of walking, which is characterised by an overall loss of physical function. Common signs and symptoms of each condition affect—or are affected by—walking performance both directly and indirectly. |
| The mental and emotional walking experience | Walking is often accompanied by feelings of fear, anxiety, stress, anger, frustration and embarrassment during or in anticipation of walking activities. Fear and anxiety often cause withdrawal and reduced participation, resulting in feelings of loneliness and hopelessness. As walking becomes increasingly effortful, additional motivation is required to initiate and complete activities. |
| The social walking experience | Individuals’ social networks influence walking experiences at home, at work and in the broader community and therefore moderate the ability to sustain participation in daily life. |
| The context of the walking experience | Physical, mental and emotional and social experiences of walking are framed within physical, social-cultural and personal contexts. Physical contexts include indoor and outdoor environments in individuals’ life spaces. Furthermore, weather and climate often make walking more dangerous or difficult. The socio-cultural context includes social and cultural norms, which determine whether individuals engage in certain walking-related activities or not. Individuals often compare themselves to others to assess and contextualise their own level of walking impairment. Finally, the personal context includes personal attitudes, past experiences, ageing, the presence of comorbidities and other characteristics that could impact the walking experience. |
| Behavioural and attitudinal adaptations resulting from the walking experience | Walking experiences frequently prompt attitudinal and behavioural responses, many of which entail strategies for coping with the effects of walking impairment. Some strategies, such as engaging in structured exercise or participating in interventions, aim to maintain function. Others aim to manage changing abilities through staying positive, planning ahead, pacing or adopting assistive technologies. |
| **Health condition specific rules** | |
| Time | Any element of time that appears to be important to people, including the influence of medication (ie within day) |
| Context | The environment they are in. For example, the impact of weather, indoor or outdoors, uphill etc. |
| Balance/unsteady/walking drunk/wobbly etc. | Coding ‘balance/unsteadyness/walking drunk’ is difficult as it could be multiple DMOs As a result, we will code it as ‘Secondary DMO’. However, we should code it as “stride length” “step duration’ and ‘speed” as well. |
| Freezing | Freezing in PD is not likely to be captured in this list of DMOs so code as ‘Secondary DMO’, especially if someone mentions freezing at the start of an activity or walk. However, if they mention freezing in certain activities (e.g. going through a door) then code this as ‘WB duration’, 'step duration' and ‘cadence’ as it might be seen within this DMO. If freezing is mentioned in MS, code it in the same way as foot-drop. |
| Foot drop | Code foot drop in MS as ‘stride length’, ‘step duration’ and ‘walking speed’. |
| Fatigue | Fatigue/tiredness is likely to be condition specific, or, it may be different in MS than others. Therefore, we may need to code this differently per condition. COPD/PFF/PF code as volume, MS code as speed and volume |
| Quality of walking/disease progression | Many participants talk about wanting to know if the quality of their walking changes, or how their condition has changed, however it is unclear what quality or change means. It was agreed that unless it is clear what they mean, then quality should not be coded as a DMO. |
