# Supplementary material for: Cocreating the Visualization of Digital Mobility Outcomes: Delphi-Type Process With Patients
Source: JMIR Form Res. 2025 May 9;9:e68782. doi: 10.2196/68782 (PMC12102624; doi:10.2196/68782)
Supplement: Multimedia Appendix 3 [file formative_v9i1e68782_app3.docx]

To develop the visualisations, we have mapped patient experiences to DMOs and drafted visualisations from the TVS data. Some of the DMOs that we have identified are important in different contexts. For example, walking speed may be important on its own, or, in relation to balance.

The table lists the topics of importance per health condition, including important time points and contextual factors. DMOs are listed in the Table according to how frequently they are listed by patients. This Table was used to identify relevant data to pull from the TVS, to develop visualisation examples.

**DMOs and their important time/contextual factors to pull from the TVS data**.

| **DMO/Group of DMOs** | **Reason for inclusion** | **Relevant time periods** | **Relevant contexts** |
| --- | --- | --- | --- |
| **PD** | | |  |
| Stride length; step duration; walking speed | Balance | Within day activities; Between day | Indoor v outdoor (or being away from home) |
| Step count | Fatigue; speak about doing less | Within day activities; Between day |  |
| Walking speed | Talk about slowing down | Within day activities; Between day |  |
| No. WB | Need to take breaks |  |  |
| WB duration; cadence | Freezing |  | Indoor v outdoor (or being away from home) |
| Stride length; step duration | Step length or shuffle |  |  |
| WB duration | How long they can walk for |  |  |
| **MS** | | |  |
| Walking speed; Step count | Fatigue | Between day | Weather; Indoor v outdoor |
| Step count | Speak about doing less | Between day | Weather; Indoor v outdoor |
| Stride length; step duration; walking speed | Balance | Between day | Weather; Indoor v outdoor |
| Stride length; step duration; walking speed | Foot drop |  | Indoor v outdoor |
| WB duration | How long they can walk for/Breaks | Between day | Indoor v outdoor |
| **COPD** | | |  |
| Step count | Fatigue; Speak about doing less | Between day; Within day | Indoor v Outdoor |
| **PFF** | | |  |
| Step count | Fatigue; Speak about doing less |  |  |
| Walking speed | Slowing down |  |  |
| Stride length; step duration | Step length |  |  |
| Stride length; step duration; walking speed | Balance |  |  |
| WB duration | Needs to take breaks |  | Indoor v Outdoor |
